# Ogilvie Syndrome Following an Inflatable Penile Implant

**DOI:** 10.7759/cureus.9279

**Published:** 2020-07-19

**Authors:** Anupam K Gupta, Joseph Farshchian

**Affiliations:** 1 Minimally Invasive Surgery, University of Miami Hospital, Miami, USA; 2 Surgery, Florida Atlantic University College of Medicine, Boca Raton, USA

**Keywords:** ogilvie, ogilvie syndrome, penile implant, obstruction, psuedocolonic obstruction

## Abstract

A 59-year-old male patient presented with Ogilvie syndrome which developed after inflatable penile prosthesis placement. The patient presented to the emergency room three days after having an inflatable penile prosthesis with complaints of obstipation. A trial of conservative measures failed, and because of the development of peritonitis, the patient underwent a right hemicolectomy with a loop ileostomy.

## Introduction

Inflatable penile prostheses (IPPs) have been a successful method for treating men with erectile dysfunction after development 30 years ago. The system consists of two intracorporeal cylinders, a scrotal pump, and a fluid reservoir placed in the abdomen, with variations in the mechanism of the pump and reservoir [1].

Ogilvie syndrome, also known as acute colonic pseudo-obstruction, is a well-known entity in patients [1,2]. Multiple situations predispose to the development of Ogilvie syndrome; however, the exact etiology is not known [3-6]. After an IPP placement, it is unusual to see the development of Ogilvie syndrome in a patient without medical comorbidity.

## Case presentation

A 59-year-old male patient with a prior surgical history of radical prostatectomy for prostate cancer underwent surgery for penile prosthesis and had an IPP implanted. The patient had no medical co-morbidities, and the last screening colonoscopy, which was done a year prior, was negative for any pathology.

**Figure 1:**
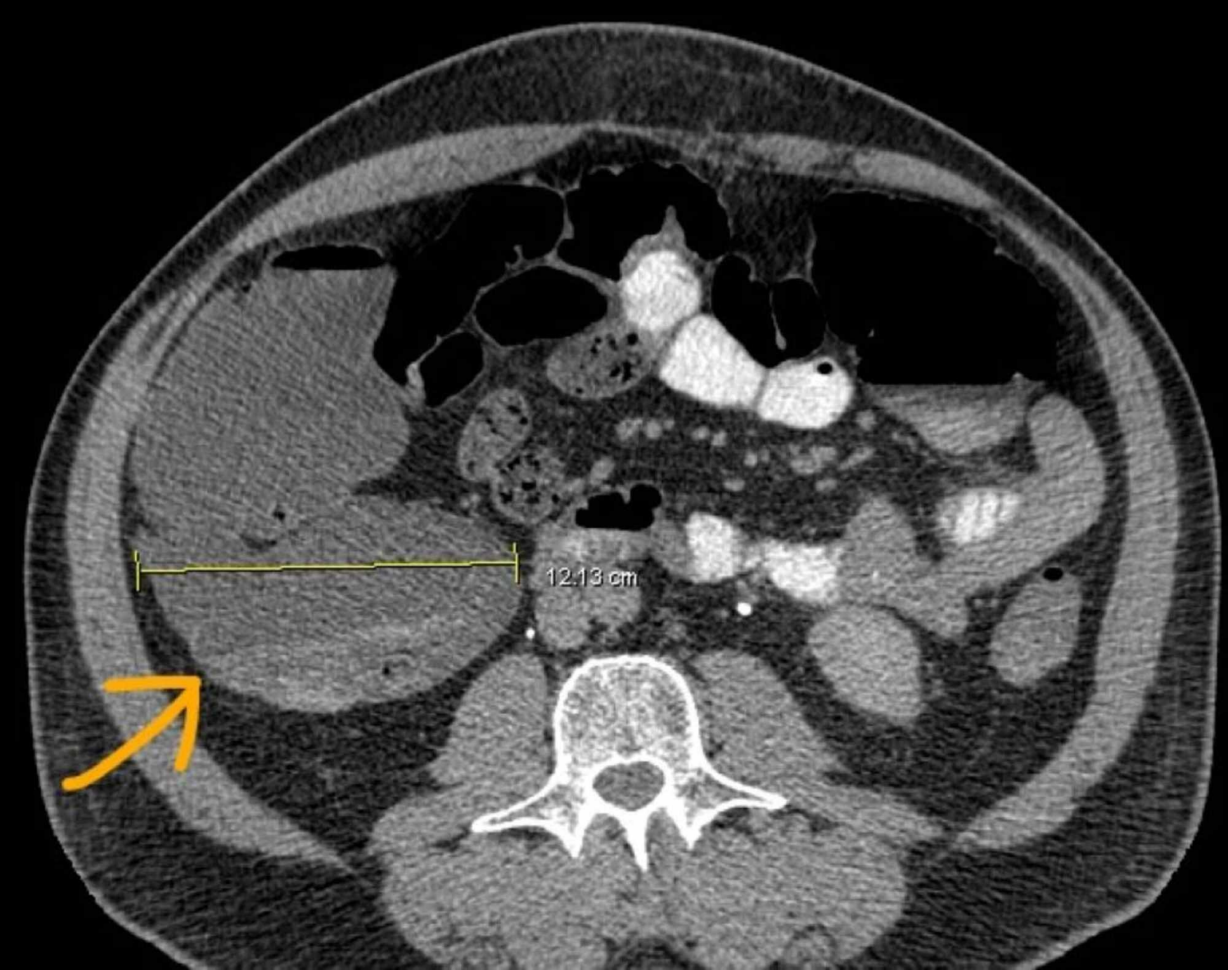
Dilated cecum 12.1 cm

**Figure 2:**
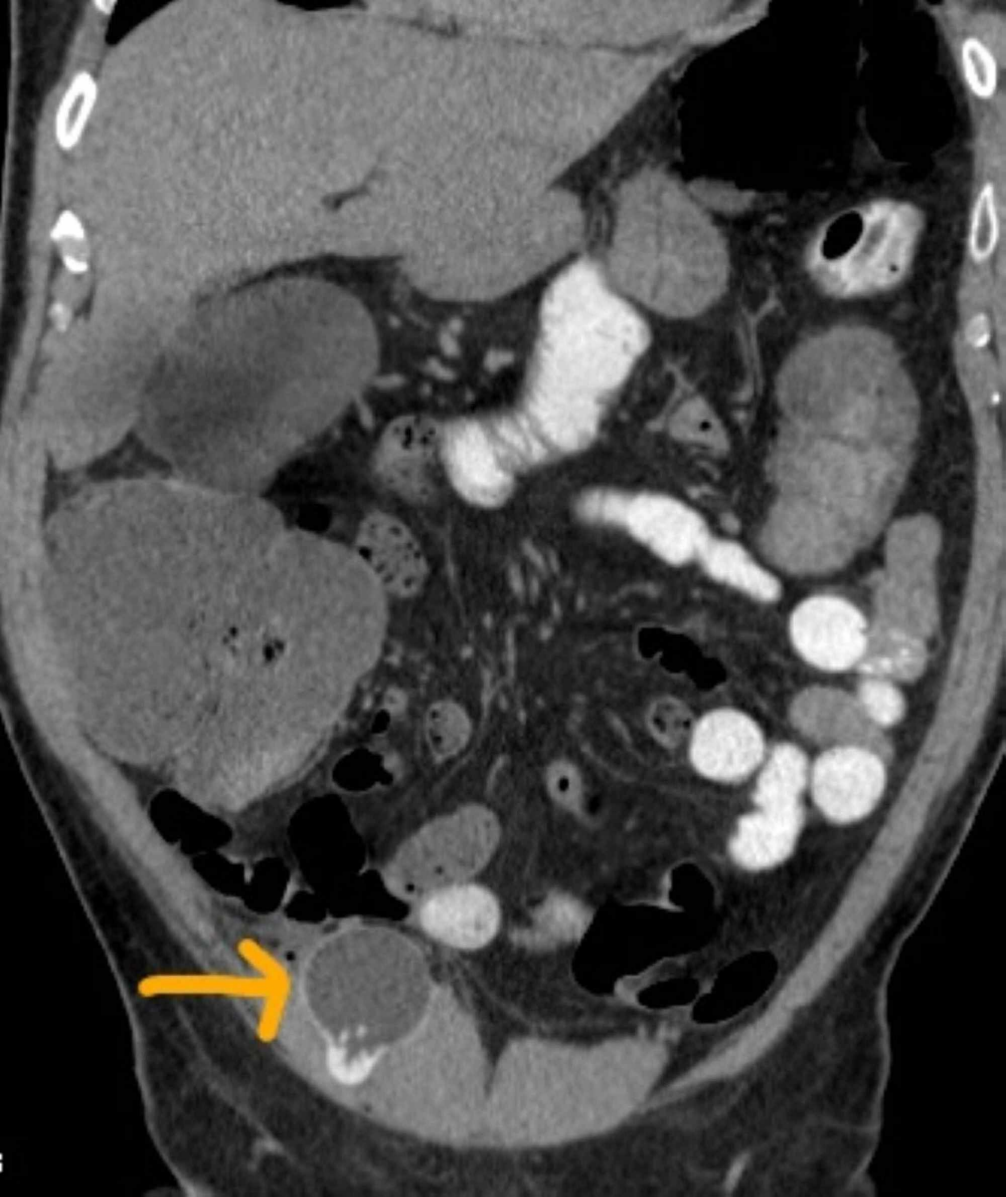
Dilated cecum with arrow showing reservoir for penile prosthesis

On postoperative day 1, the patient developed constipation and bloating, which were not relieved by over-the-counter Fleet enemas or laxatives that the patient purchased. To note, the patient had not been receiving opioids for analgesia. Since the symptoms had persisted and worsened, the patient returned to the hospital. On initial evaluation, the patient had vital signs within normal limits and physical findings remarkable for abdominal bloating only. A complete blood count and basic metabolic panel were within normal limits. CT of the abdomen and pelvis revealed a dilated cecum (12.1 cm) with no obstructive pathology (Figures 1, 2). The patient was subsequently admitted and started on a conservative management regime, including bowel rest, intravenous maintenance fluids, and pain control without narcotics. Eight hours postadmission, he complained of increasing abdominal pain. The patient was re-examined and displayed evidence of peritonitis.

The patient underwent emergency exploratory laparotomy, which revealed an ischemic cecum with massive dilatation, and he was treated with right hemicolectomy with a loop ileostomy. The penile prosthesis appeared to be in the desired position with no pathology.

The postoperative course was uneventful, and the patient went home on postoperative day 7. The patient had a reversal of loop ileostomy after two months with no complications.

## Discussion

Ogilvie syndrome is an acute colonic pseudo-obstruction that consists of the dilatation of either part or all of the colon and rectum, without intrinsic or extrinsic mechanical obstruction [7]. Typically, Ogilvie syndrome occurs in hospitalized patients with severe illness or trauma or following general, orthopedic, neurosurgical, or gynecological procedures [8]. The incidence is estimated to be 0.1% of hospital admissions, with a mortality rate of 8%. Furthermore, the feared complications of colonic ischemia and perforation may occur in up to 15% of these patients, with an estimated 40% mortality.

The pathophysiology remains poorly understood. What is known is that the syndrome is a result of an imbalance in colonic autonomic innervation. Ogilvie was the first to theorize the “sympathetic deprivation” of the colon as the likely mechanism involved in the diagnosis [9]. The leading theory suggests that there is a relative excess of sympathetic tone over parasympathetic tone. Ogilvie’s syndrome is likely a result of reduced parasympathetic innervation to the distal colon, which causes atony and essentially a functional obstruction [10,11].

Although the development of Ogilvie syndrome is associated with a variety of procedures, it is rare to occur after the placement of an IPP. Typically, placement of an IPP is indicated for erectile dysfunction refractory to medical treatment and to treat Peyronie’s disease, prolonged priapism, and phalloplasty [12]. In standard practice, the all-cause complication rate is less than 5% [13]. Complications include hematoma, floppy glans, corporal fibrosis, corporal perforation, urethral injury, infection, impending erosion, and glandular ischemia. A case of Ogilvie’s syndrome associated with the placement of an IPP is unusual.

The management of Ogilvie syndrome is a stepwise approach that consists of conservative management, medical interventions, or surgery, depending on the severity and extent of colonic dilation [14,15]. Conservative measures include giving the patient intravenous fluids, not allowing the patient to given anything by mouth, decompression of the gastrointestinal tract by nasogastric tube, and encouraging the patient to ambulate if possible. If colonic dilation accrues to greater than 12 cm, as in our patient’s case, neostigmine, an acetylcholinesterase inhibitor, may be tried. Due to the acuity of the case and the quick development of peritonitis, neostigmine was not used, and surgical intervention was necessary. In our case, the patient underwent an exploratory laparotomy, revealing an ischemic cecum with massive dilatation that required a right hemicolectomy with a loop ileostomy that was reversed two months later.

## Conclusions

Ogilvie syndrome is a rare but known condition with direct etiology still unknown. We report an unusual case of a patient with Ogilvie syndrome where radical surgical treatment was necessary to treat their developing peritonitis. 
